# Survey of bacteria associated with western corn rootworm life stages reveals no difference between insects reared in different soils

**DOI:** 10.1038/s41598-019-51870-x

**Published:** 2019-10-25

**Authors:** Dalton C. Ludwick, Aaron C. Ericsson, Lisa N. Meihls, Michelle L. J. Gregory, Deborah L. Finke, Thomas A. Coudron, Bruce E. Hibbard, Kent S. Shelby

**Affiliations:** 1 USDA-ARS, 2217 Wiltshire Rd., Kearneysville, WV 25430 USA; 2 Department of Veterinary Pathobiology, University of Missouri, Columbia, MO 65201 USA; 3 USDA-ARS, Donald Danforth Plant Science Center, St. Louis, MO 63132 USA; 4 USDA-ARS, 1503 S. Providence Rd., Columbia, MO 65203 USA; 5 Division of Plant Sciences, University of Missouri, Columbia, MO 65211 USA; 6 USDA-ARS, 205 Curtis Hall, University of Missouri, Columbia, MO 65211 USA

**Keywords:** Biogeochemistry, Bacteria

## Abstract

Western corn rootworm (*Diabrotica virgifera virgifera* LeConte) is a serious pest of maize (*Zea mays* L.) in North America and parts of Europe. With most of its life cycle spent in the soil feeding on maize root tissues, this insect is likely to encounter and interact with a wide range of soil and rhizosphere microbes. Our knowledge of the role of microbes in pest management and plant health remains woefully incomplete, yet that knowledge could play an important role in effective pest management strategies. For this study, insects were reared on maize in soils from different locations. Insects from two different laboratory colonies (a diapausing and a non-diapausing colony) were sampled at each life stage to determine the possible core bacteriome. Additionally, soil was sampled at each life stage and resulting bacteria were identified to determine the possible contribution of soil to the rootworm bacteriome, if any. We analyzed the V4 hypervariable region of bacterial 16S rRNA genes with Illumina MiSeq to survey the different species of bacteria associated with the insects and the soils. The bacterial community associated with insects was significantly different from that in the soil. Some differences appear to exist between insects from non-diapausing and diapausing colonies while no significant differences in community composition existed between the insects reared on different soils. Despite differences in the bacteria present in immature stages and in male and female adults, there is a possible core bacteriome of approximately 16 operational taxonomic units (*i.e*., present across all life stages). This research may provide insights into Bt resistance development, improved nutrition in artificial rearing systems, and new management strategies.

## Introduction

The western corn rootworm (*Diabrotica virgifera virgifera* LeConte; WCR) is a chrysomelid beetle whose larvae cause damage to maize root systems. While native to North America, this pest was introduced multiple times to Europe over 20 years ago^1^. Most recent estimates indicate this pest causes $2B (USD) in yield loss plus control costs worldwide annually^2,3^. Since its discovery as a pest of maize, the primary control tactic has been crop rotation^4^. Recently, transgenic maize hybrids expressing pore forming entomotoxic δ-endotoxins from *Bacillus thuringiensis* Berliner (Bt) have been used to reduce root damage and economic losses. However, both crop rotation and transgenic strategies have instances of failure in the U.S. Corn Belt^5–10^.

Neonate rootworm larvae (WCR and *D. barberi* Smith & Lawrence) burrow through the soil searching for maize roots where they feed on root cortex tissue^10^. Thus, after hatching with a presumably sterile, uncolonized gut, larvae of these species navigate within a dynamic species-rich maize-conditioned rhizosphere and come into direct oral contact with the maize phylloplane during immature developmental stages. Larvae pupate in the soil and adults emerge to damage maize silks and leaf tissue, thereby additionally coming into contact with microbes of the above-ground maize phyllosphere. Finally, adults are known to fly many kilometers from their larval home to infest distant maize fields^10^ where the microbial community composition they and their offspring encounter may be discordant. In WCR adults, exposure to diverse bacterial species would occur mainly through cuticular surfaces and within their digestive tracts. Throughout development, WCR larval holobionts would be expected to reflect the diverse microbial assemblies particular to the soils and rhizospheres they encounter. In addition to these perhaps transient environmental exposures, larvae may possess maternally transmitted microbiota^11^.

Insect gut microbiomes are known to influence many aspects of growth, nutrition, reproduction, Bt resistance, and pathogen resistance^11–19^. Gut microbiota have been shown to affect the response of insects to Bt proteins in Lepidoptera^13,17–19^ and in mosquitoes^20^, but this has not been investigated for Coleoptera. Gut microbiota are also required for susceptibility of the gypsy moth, *Lymantria dispar* (L.), to Bt proteins^13^. In the Old World bollworm (*Helicoverpa armigera* Hübner), the manipulation of the larval gut microbiota with antibiotics resulted in reduced susceptibility to a commercial formulation of Bt, as well as the purified δ-endotoxins Cry1Ab and Cry1Ac^17^. In general, the use of antibiotics to manipulate lepidopteran gut microbiota resulted in reduced mortality due to Bt proteins. Selection experiments with *H. armigera* on transgenic plants were also conducted in addition to manipulation of gut microbiota with antibiotics^19^. When antibiotics were included, susceptibility to Bt was not altered with increasing generations of selection. However, selection in the absence of antibiotics (gut microbiota unaltered) resulted in a nearly 30% increase in larval survival by the F3 generation^19^. Thus, resistance to Bt by *H. armigera* developed only in the absence of antibiotic treatment. In fact, the reduction in susceptibility to Bt with the addition of antibiotics was greater than the reduction of susceptibility to Bt due to three generations of selection when gut microbiota were present. Interestingly, Lepidoptera appear to lack a stable microbiome, either because of the high flux of plant material through the gut, high pH, or other factors^21^. This was especially evident when measures were taken to exclude dead bacteria by amplifying from rRNA^22^.

Several studies have evaluated the microbial communities associated with lepidopteran pests and other insects that attack food crops^14,23–25^. However, fewer studies have been conducted to document microbiomes within beetles attacking field crops. Within the *Diabrotica* species, *D. speciosa* (Germar), *D. balteata* LeConte, *D. undecimpunctata howardi* Barber, and WCR have been studied. *D. speciosa* is a polyphagous pest attacking several important crops in South America including corn^26^, *D. balteata* is a pest of sweet potato^26^, and *D. undecimpunctata howardi* attacks cucurbits and corn^26^. Shalk *et al*. (1987), through culturing, identified bacteria from larvae, field collected adults, and laboratory reared adults of *D. balteata*. Tran and Marrone (1988), again through culturing, identified bacteria present in the guts of first instar larvae of *D. undecimpunctata howardi*. Chu *et al*. (2013), through culture independent methods, identified gut bacteria in WCR which facilitate adaptation to crop rotation. Most recently, Perlatti *et al*. (2017) used culturing followed by 16S and matrix-assisted laser desorption ionization coupled to time-of-flight mass spectrometry to identify gut bacteria in *D. speciosa*. Combining data from these species, Perlatti *et al*. (2017) were able to identify a “core” gut microbiome for the genus *Diabrotica*.

WCR has been studied more intensely than other *Diabrotica* species in recent years. Larval gut tissue of *Diabrotica* larvae have a diverse microbial community^15,27^. In WCR, an increase in some bacterial taxa (e.g. *Acinetobacter* sp., *Pseudomonas* sp.) and decrease in others (e.g. *Enterobacter* sp., *Lactococcus* sp.) within the gut microbiota profile was associated with increased resistance to soybean defense compounds, which may have contributed to the development of resistance to crop rotation^27^. Comparison of gut microbiota between rotation-resistant WCR populations and wild-type WCR populations revealed a seven-fold difference in *Klebsiella* sp. and *Stenotrophomonas* sp. abundance and two-fold difference in *Enterobacter* sp., *Lactococcus* sp., and *Enterococcus* sp. between rotation-resistant and wild-type WCR. Manipulation of rotation-resistant WCR gut microbiota with antibiotics reduced the resistance to soybean defensive compounds to a level similar to that of wild- type WCR^27^. While the researchers have primarily focused on bacterial components, an insect microbiome may also contain fungi, viruses, archaea, and protozoa^28^. Preliminary studies have identified at least three viruses present in WCR^29–31^.

Contributions of the microbiota to the biology of WCR and its importance in insecticide and Bt resistance are just now being elucidated^15^. WCR larval feeding on maize root tissue was shown to affect root rhizoplane microbial composition, indicating a complex, multi-trophic interaction^16^. Since midgut microbiota play a role in Bt susceptibility in lepidopteran pests and is also involved in WCR resistance to crop rotation, it is reasonable to hypothesize that WCR microbiota can affect how larvae respond to maize-expressed Bt toxins. We further hypothesize that discordant locality-specific rhizosphere microbial assemblages may contribute to observed spatial variation in WCR susceptibility to Cry3 Bt toxins^32^. In this study, we evaluated bacterial populations in two WCR colonies (a non-diapausing colony and a diapausing colony) at each developmental stage. We also reared insects of the non-diapausing colony on two different soil types and evaluated bacterial populations at each developmental stage. Soil samples from each test environment were also evaluated. We demonstrate that WCR can carry particular bacterial species across all life stages (*i.e*., a core bacterial populations) regardless of the rearing environment.

Discordant rhizosphere species assemblages present in adjacent or distant maize fields could affect the microbiota encountered by developing larvae, and this may be reflected in observation of discordant microbiomes within feeding WCR. Microbiota encountered as the larvae traverse soils to the rhizosphere and pierce the phylloplane to feed also may converge on a concordant assemblage^33^. Concordant rhizosphere assemblages would be expected to result in no discernable differences in sampled WCR microbiomes. Consequently, a better understanding of which microbes are associated with WCR and how the insects acquire their microbiome is needed.

## Results and Discussion

We conducted a survey of the bacterial assemblages in two laboratory populations of WCR (non-diapausing and diapausing) across all life stages from egg to adult. In addition, we surveyed the soil in which the insects had developed. Because WCR occurs across a large region, and in many different soils throughout the United States of America and Europe, we also investigated the effect of soil geographic origin on the resulting WCR bacterial assemblages. To accomplish this, we collected soil from Higginsville, MO, and used this soil to perform the same life stage survey/soil survey using the diapausing colony. The Higginsville, MO, site had been planted with maize in the year of soil collection and in previous years to increase the WCR population. This survey allows us to compare bacterial assemblages (1) between every life stage, (2) between non-diapausing and diapausing colonies, (3) between autoclaved and field soil, and (4) between the insect itself and the soil in which it developed.

Sequencing of bacterial populations in WCR and soil samples resulted in a mean (±SEM) of 66,759 (±3,895) and 72,868 (±5,308) reads per sample, respectively (Table S1). To account for the potential influence of differential coverage on downstream analyses, data were randomly subsampled to a uniform depth of 10,000 reads per sample and all subsequent analyses were performed on this rarefied dataset (Table S1). The results show that earlier life stages reared in soils from different locations contained a significantly discordant assemblage of bacterial species. However, as the insects matured, discordance declined and all life stages converged to a similar bacterial assemblage.

**Figure 1:**
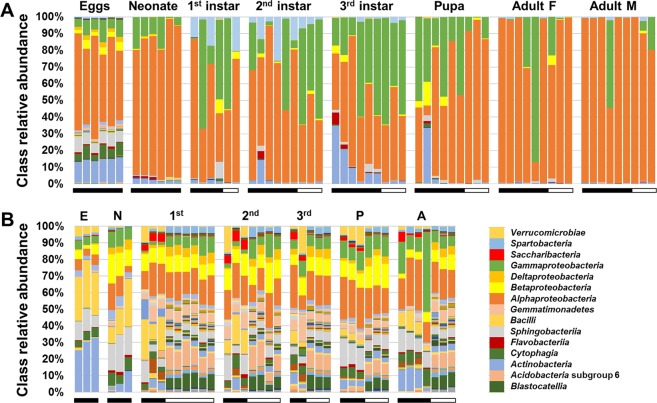
Stacked bar charts showing relative abundances of bacterial classes detected in WCR at different life stages (**A**) and in soil from which WCR samples were collected (**B**). Horizontal bars below the vertical bars indicate origin of soil; black bars = Columbia, MO, white bars = Higginsville, MO.

Annotated to the taxonomic level of class, the WCR samples were dominated by *Alphaproteobacteria* and *Gammaproteobacteria*, with lower and inconsistent relative abundance of *Actinobacteria*, *Cytophaga*, *Sphingobacteria*, *Betaproteobacteria*, and in the case of surface-sterilized eggs, *Flavobacteriia* and *Deltaproteobacteria* (Fig. 1A). Soil samples had a seemingly more complex microbiota comprising a greater number of bacterial classes and a more even distribution (Fig. 1B).

Microbial richness and diversity are often correlated with the health of an ecosystem, be it environmental or host-associated^26^. Richness simply denotes the overall number of detected phylotypes in a sample, whereas the Shannon and Simpson diversity indices integrate both the richness and evenness of the distribution of phylotypes in a sample^34^. The underlying assumption is that increased numbers of different taxa and more even distributions of those taxa are representative of ecosystems fostering cross-feeding and syntrophic relationships among microbes. In contrast, low richness or asymmetrical distributions might represent an environment with high selective pressures or the presence of dominant taxa in a competitive environment^34^.

Analyses of richness and diversity of bacterial communities in WCR and in the soil in which they had developed revealed several interesting trends (Table S1). To first determine whether the geographic site of soil origin influenced richness, Shannon diversity index, or Simpson diversity index of WCR bacteria, a two-way ANOVA was performed with soil site (*i.e*., Columbia or Higginsville) and insect life-stage as fixed variables. Significant main effects of WCR life-stage were assessed for richness (*p* < 0.001, n = 63, F = 8.14), Shannon index (*p* = 0.011, n = 63, F = 3.48), and Simpson index (*p* < 0.001, n = 63, F = 5.78). No differences were detected between soil sites for richness, Shannon index, or Simpson index of WCR-associated bacteria (*p* = 0.338, n = 41, 0.072, and 0.244, respectively). Of note however, similar testing of the soil communities from each site revealed significant site-dependent differences in richness, Shannon index, and Simpson index (*p* < 0.001 for all three metrics, n = 41, F = 38.52, 197.64, and 25.04, respectively). No differences in bacterial richness were detected between the two soil collection sites over the larval life stages, although diversity within the soil bacterial assemblage did vary significantly among life-stages (*p* = 0.030, n = 63, F = 2.88 and *p* < 0.001, F = 5.53 for Shannon and Simpson indices, respectively).

Collectively, these data suggest that the environmentally available bacteria have a limited effect on the relative uniformity and richness of the WCR bacterial assemblages. This conclusion is supported by the nearly log-fold difference in richness between soil and WCR samples and the fact that no soil-dependent differences were detected in the bacterial assemblages of WCR despite the stark differences in the bacterial richness of their respective environments.

**Figure 2:**
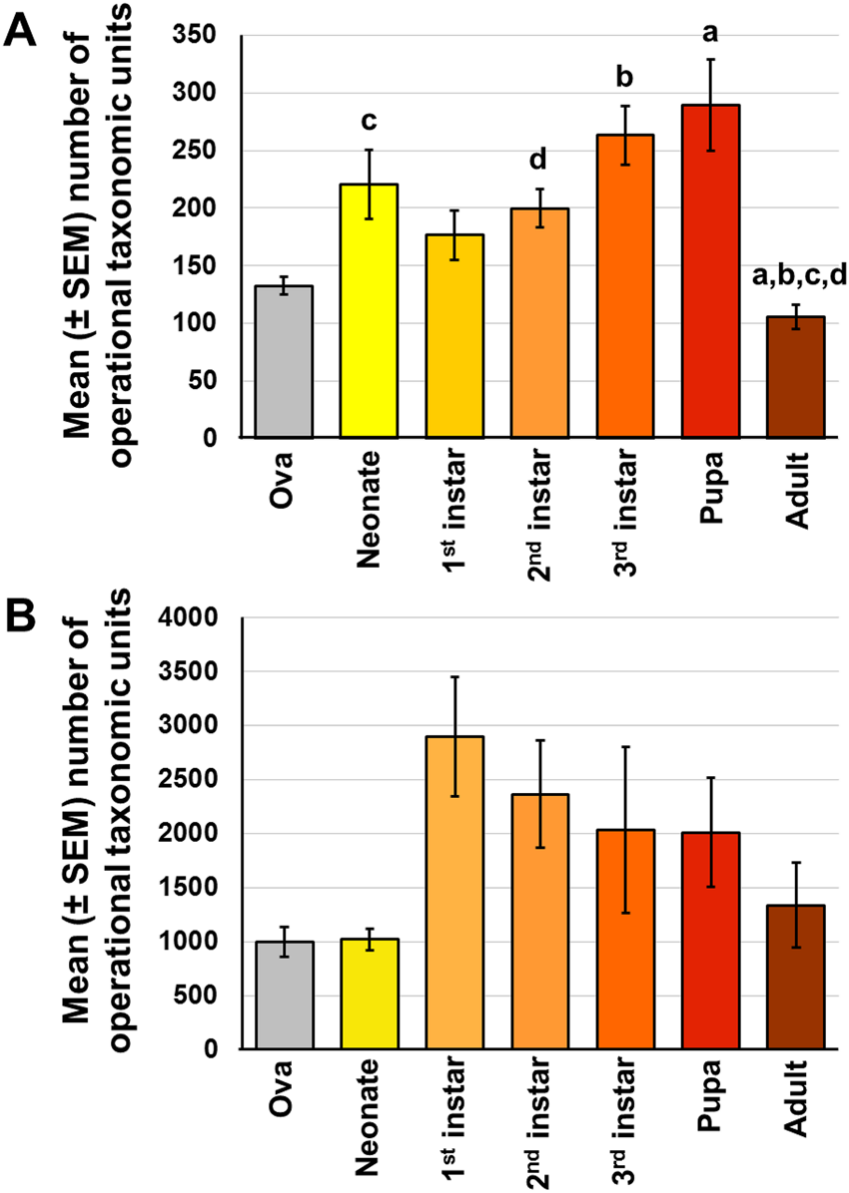
Main effect of life stage on bacterial richness in WCR (**A**, *p* < 0.001), or the soil from which WCR samples were collected (**B**, *p* = 0.040). Significant pairwise differences are indicated by like letters (Kruskal-Wallis one-way ANOVA on ranks with Dunn’s *post hoc*).

Considering WCR samples from the two soils collectively, there was a general trend toward increasing richness in each successive life-stage from egg to pupa followed by a precipitous decline during the pupal molt to adulthood (Fig. 2A). Pairwise comparisons of richness between life-stages detected significantly decreased richness of phylotypes in adult WCR relative to several earlier life-stages. Interestingly, an inverse trend was observed in the richness of bacteria in soil samples across life-stages (Fig. 2B). In contrast, diversity as assessed via the Simpson index, was higher in sterilized eggs relative to other life-stages while diversity in adult WCR was much lower (Fig. S1A**)**, likely reflecting the increasingly skewed bacterial community structure as the WCR mature. No life-stage-dependent differences were detected in the diversity of the soil bacterial community (Fig. S1B).

In order to provide a more comprehensive comparison among the bacterial communities present in each sample, principal coordinate analysis (PCoA) and permutational multivariate analyses of variance (PERMANOVA) were performed to visualize and statistically test for differences in community structure, respectively. With both methods, the similarity of any given pair of samples can be determined in several different ways. To ensure that any differences detected were robust and to determine the nature of detected differences, we compared samples using both the Bray-Curtis and Jaccard similarity indices. While the Jaccard index is relatively unweighted and determines sample similarity based on the shared presence or absence of taxa, the Bray-Curtis index is weighted and incorporates the relative abundance of any shared taxa.

**Figure 3:**
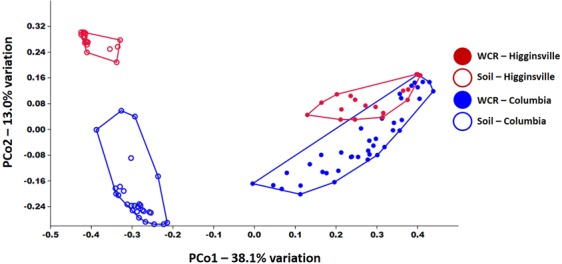
Principal coordinate analysis based on Bray-Curtis similarity between bacterial communities detected in WCR at various life stages and soil samples collected from two different sites.

Regardless of the index used, robust compositional differences were detected among all groups with the exception of the WCR samples reared in soil from different sites, again suggesting selection for a specific bacterial community within the WCR. Specifically, testing for differences using the Bray-Curtis distances detected significant compositional differences between all pairwise comparisons except between WCR samples reared in different soil (Table 1). Accordingly, PCoA demonstrated a clear separation of soil and WCR samples along PC1 (38.1% of the total variation in the dataset), complete separation of soil communities from the two soil sites along PC2, and partial overlap between WCR communities (Fig. 3). Testing based on the Jaccard index found significant differences between all pairwise comparisons. Ordination resulted in a similar pattern and the F value generated from the comparison of WCR reared on soil from the two sites was extremely low relative to the other comparisons, despite having the highest total number of samples included in the comparison (Table 2). Together, these data complement the analyses of richness and diversity in supporting the hypothesis that WCR select for a limited subset of host-associated bacteria, largely irrespective of their environment.

**Table 1:** Results of PERMANOVA testing for differences in β-diversity between WCR and soil samples collected from two different sites, based on the Bray-Curtis distance. *p* values and F values are shown in the upper right and lower left portions of the table, respectively.

*p* values		Soil origin		WCR from “X” soil	
F values		Columbia	Higginsville	Columbia	Higginsville
Soil origin	Columbia		0.0001	0.0001	0.0001
	Higginsville	27.62		0.0001	0.0001
WCR from “X” soil	Columbia	57.08	104.5		0.1498
	Higginsville	38.43	119.7	1.657

**Table 2:** Results of PERMANOVA testing for differences in β-diversity between WCR and soil samples collected from two different sites, based on the Jaccard distance. *p* values and F values are shown in the upper right and lower left portions of the table, respectively.

*p* values		Soil Origin		WCR from “X” soil	
F values		Columbia	Higginsville	Columbia	Higginsville
Soil Origin	Columbia		0.0001	0.0001	0.0001
	Higginsville	24.93		0.0001	0.0001
WCR from “X” soil	Columbia	19.62	23.66		0.0001
	Higginsville	18.56	18.6	3.972	

Annotated to the level of operational taxonomic unit (*i.e*., the best taxonomic resolution afforded by the 16S rRNA amplicons), the bacterial composition of the adult WCR was incredibly sparse. Of the 474 operational taxonomic units (OTUs) detected in anywhere from one (5.6%) to 15 (83.3%) of 18 adult WCR, the mean relative abundance was uniformly below 0.3% (Fig. S3). Conversely, the 13 OTUs detected in 16 or greater of the 18 adult WCR were present at a mean relative abundance of greater than 1.5%. Notably, 95.4% of the bacterial DNA recovered from adult WCR was annotated to three OTUs: *Wolbachia* sp. (85.5 ± 24.0% in 18 of 18 adults), unclassified family *Enterobacteriaceae* (6.2 ± 13.0% in 16 of 18 adults), and *Acinetobacter* sp. (4.7 ± 11.6% in 17 of 18 adults).

**Figure 4:**
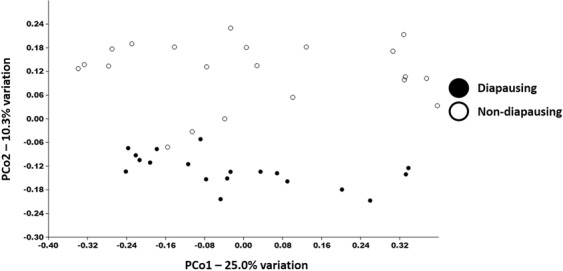
Principal coordinate analysis based on Bray-Curtis similarity between bacterial communities detected in WCR from diapausing and non-diapausing colonies including all life stages, except surface sterilized eggs.

To determine whether different genetic backgrounds could lead to different bacterial assemblages in WCR, the present study we reared insects from both a colony of diapausing WCR and a non-diapausing WCR laboratory colony in autoclaved soil from Columbia, MO, as previously described. Once the identities of the bacteria were determined, we compared the bacterial assemblages of the two colonies using PERMANOVA with Bray-Curtis and Jaccard indices. The two indices revealed different patterns. No significant differences were detected between these colonies with the Bray-Curtis index (*p* = 0.10; 43, F = 1.90), indicating that insects that are non-diapausing retain a bacterial assemblage similar to insects that diapause, despite hundreds of generations of laboratory selection. However, PERMANOVA with a Jaccard index revealed significant differences in bacterial assemblages between non-diapausing and diapausing insects (*p* = 0.0001; 43, F = 2.90; Fig. 4). Insects from both colonies appear to share many dominant taxa while rarer species appear to be unique to individual colonies.

Exploratory studies documenting bacterial assemblages in different organisms may lead to new insights into the role(s) they may fill or even new management tactics. Over 2,200 unique operational taxonomic units (OTUs) were putatively identified in soil and insect samples from both colonies and soils. Our study documented more than 1,100 OTUs present throughout the WCR life cycle. Of these OTUs, 16 were found in every life stage of the insect regardless of the colony or rearing soil. We speculate that these 16 OTUs comprise a core bacterial assemblage for WCR. Furthermore, six of these bacterial species were found in the insect sample but not the soil sample (Table 3). *Wolbachia*, a known WCR endosymbiont^35^, was detected in the soil samples collected when the insects were neonates. It is possible that they may have originated from the hatching eggs. Vertical transmission (*i.e*., parent to progeny) of bacteria is the most likely mechanism for at least some of the WCR bacteriome as some bacteria were never found in soil samples^35^ (Table 3).

**Table 3:** Unique operational taxonomic units (OTUs) found in all insect samples regardless of soil origin.

OTUs	Taxonomic Rank^b^	Present in egg soil?^a^	Corresponding insect life stage?^c^
Ruminococcaceae	Family	Yes	Egg
Lachnospiraceae	Family	Yes	Egg
Bacteroidales S24-7	Group	Yes	Egg
*Wolbachia* (*Delia antiqua*)	Genus	No	Neonate
*Tsukamurella* sp.	Genus	No	—
*Gordonia* sp.	Genus	Yes	Egg
*Oscillibacter* sp.	Genus	Yes	Egg
*Microbacterium* sp.	Genus	Yes	Egg
*Bacillus megaterium* ^c^	Genus	No	—
*Geobacillus toebii*	Genus	Yes	Egg
*Klebsiella* sp. Z1	Genus	Yes	Egg
*Mycobacterium fortuitum*	Genus	No	—
*Streptomyces rectiviolaceus*	Genus	No	—
Lachnospiraceae NK4A136	Genus	Yes	Egg
*Pseudomonas* sp. FSGRN7	Genus	No	—
*Pseudonocardia* sp. YIM 68245	Genus	No	—

^a^Were the OTUs found in the soil in which eggs were incubated and neonates emerged?

^b^Taxonomic annotations beyond the level of genus represent the closest sequence match in the SILVA database but do not necessarily imply accurate species-level identification.

^c^If these OTUs were found in soil samples, then at which corresponding insect life stage were these OTUs first detected in soil samples?

Available microbial sampling and survey methods have known and unknown flaws. Most early publications of *Diabrotica* associated bacteria relied on bacterial culture followed by morphological and sequence identification^36–38^. With soil and rhizosphere bacteria, the percentage of culturable bacteria is believed to be ~1%^26^. Culture independent methods, which rely solely on DNA present in the sample, can capture these bacterial species previously undetected by earlier methods. However, culture independent DNA based methods are incapable of distinguishing dead *vs* live bacteria. Thus, although many OTUs were identified in the surface-sterilized eggs of the diapausing colony, with the DNA based method used, we cannot be certain whether the sequences originated from viable bacteria within the egg. It is equally possible that the sequences originated from dead cells attached to egg chorion surfaces. Given the intricate sculpturing of the chorion, it is possible dead bacteria remaining on the surface could have served as a source of non-viable DNA^16,39^. If the bacteria were viable, then it is possible the eggs serve as a source of bacteria to inoculate the neonatal gut post-hatching, as with other insect species^40^. Future experiments involving the extraction of rRNA and sequencing of the cDNA will help reduce the influence of dead bacteria^41^.

While the WCR bacterial assemblage was the target of this experiment, the presence of corn microbial assemblages cannot be ignored. For most of the life stages examined, corn tissue would have been present in the insect gut at the time of sampling. Corn itself is known to harbor several genera identified in our samples, including *Klebsiella*, *Pseudomonas*, and *Streptomyces*^42–44^. Additionally, since these insects were not surface sterilized prior to DNA extraction, bacterial species present on the insect cuticle will be included. While the cuticular bacterial assemblage has not been investigated in WCR, in bark beetles of the genus *Dendroctonus* several genera of bacteria including *Pseudomonas*, *Serratia*, and *Yersinia* have been identified^45^. Though not the target of the current study, cuticular surface bacterial species would nonetheless be interesting to identify given that there are instances of gut microbiota and cuticular pathogens interacting in insects^46^.

The geographic distribution of this pest insect encompasses most of the United States and parts of Europe. The soils across these regions are also diverse, as are the management tactics employed. Our research indicates that, though WCR are exposed to diverse bacterial assemblages in the soil in which they emerge and live, a small core bacterial assemblage is retained by WCR. This core bacterial assemblage has commonality with those of other *Diabrotica* species which have been examined. Additionally, diversity of bacterial assemblages decreased as the insect proceeded through life stages, culminating in adult insects which had relatively homogenous bacterial assemblages.

## Materials and Methods

### Egg source

Eggs from non-diapausing and diapausing colonies of WCR were obtained from the Agricultural Research Service of the United States Department of Agriculture (USDA-ARS). The diapausing colony eggs were from the primary diapausing colony also held at Brookings, SD, and remained in cold storage until needed^47^. The non-diapausing colony was derived from the primary non-diapausing colony held at Brookings, SD and subsequently maintained in Columbia, MO. Adults of the non-diapausing colony were reared under standard conditions prior to this study^48^.

### Egg collection – non-diapausing colony

For both the diapausing and the non-diapausing colony maintained in Columbia, MO, adult females oviposit eggs in soil filled Petri dishes (Hogentogler & Co. Inc, Columbia, MD). Petri dishes (100 × 25 mm polystyrene) were filled with ~35 ml of 70 mesh sieved soil. Approximately 30 ml of tap water is then added to the soil and the Petri dish is shaken/tapped to level the soil surface. Any excess water on the surface of the soil is absorbed by the addition of more soil. The soil is then scored several times with a knife to create furrows. Petri dishes are then covered with a lid in which four small holes have been drilled around the edge. The oviposition site was moistened throughout the week and replaced weekly.

### Egg recovery – all colonies

For collection, non-diapausing eggs were separated from the soil by first using tap water to wash soil and eggs through a 60-mesh sieve (Hogentogler & Co. Inc, Columbia, MD). The eggs were then washed from the sieve surface into a 100 ml beaker, again using tap water. Eggs were then triple rinsed with tap water to float away hatched larvae and egg cases. Healthy eggs, which sink in tap water, were then collected using a disposable transfer pipette and placed into two plastic containers (15 × 10 cm, GladWare^®^, The Glad Products Company, Oakland, CA). Eggs were covered with 70 mesh sieved Columbia, MO, field soil. The plastic containers were covered with lids and incubated at 25 °C with a photoperiod of 14:10 (L:D) until hatch.

Diapausing eggs were pulled from refrigeration and incubated at 25 °C until hatch to terminate diapause and initiate further development. As the eggs reached hatch, eggs were recovered from the incubation soil as described previously.

Sterilized eggs mentioned above were achieved using methods described in Ludwick *et al*. (2018). Sterilized eggs were then placed in 1.5 ml microcentrifuge tubes for DNA extraction as described in later sections.

### Insect development - non-diapausing colony

Each seedling mat contained approximately 15 g of maize seed (Monsanto Company, variety DKC 61–79), 6 cm of autoclaved growth medium, and 80 ml of tap water in a 15 × 10 cm plastic container. The growth medium consisted of a mixture of field soil:Pro-Mix BX potting medium (Premier Horticulture Inc., Quakertown, PA) at a 2:1 ratio (v/v) prior to being autoclaved. Seedling mats were allowed to germinate, and coleoptiles emerged through the soil surface prior to infestation. Twenty-five larvae were transferred from the egg incubation container to the seedling mats with a fine paintbrush to the soil surface. The paintbrush was sterilized with ethanol and allowed to air dry between each seedling mat.

For the adult emergence time point in this survey, we planted new maize seeds into a larger container (33 × 19 cm, Sterilite Corporation, Birmingham, AL) and allowed the maize to grow for one week prior to infestation. The first and smaller seedling mat had plant tissue removed before being inverted onto the second and larger seedling mat containing soil from the same site. After one week, the larger seedling mat was covered with a mesh screen to prevent escape of emerging adults.

### Insect development - diapausing colony

During this survey, two different growth media were used. The first growth medium remained the same as the previous insect survey, while the second growth medium was soil collected from a continuous maize field in Higginsville, MO, in July 2016. This soil was not autoclaved and remained enclosed in a metal container until use in October 2016. In addition to the time points listed previously, two types of eggs were sampled: eggs washed from sieved soil, and eggs washed from sieved soil that were then surface sterilized^49^.

No secondary container was used for the diapausing insect survey, but mesh screens were used to keep the adults from escaping the container.

### Experimental design

For both colonies, insects were collected at six time points: 0 d (neonate larvae <24 h old), 5 d, 10 d, 15 d, 22 d, and adult emergence. For the non-diapausing colony, replicates consisted of pooled individuals (1–8 larvae/treatment; 1–2 pupae/treatment; a single adult/treatment) and there were three replicates per time point. For the diapausing colony, replicates consisted of pooled individuals (1–8 larvae/treatment; 1–2 pupae/treatment; a single adult/treatment) and there were five replicates per time point. Seedling mats in each replicate were randomly assigned a time point. The 0 d time point did not require insect feeding, so 20 neonate larvae were collected immediately.

### Sample collection

Once the desired time point was reached, larvae were recovered from the seedling mats^48^. For the 5, 10, 15, and 22 d time points, all aboveground plant material was cut and removed prior to placement in the Berlese funnel. Next, the soil and root tissue were placed into a Berlese funnel which was fitted with an attached collection jar. The jar contained moistened filter paper at the bottom where larvae were collected. Specimens of each age were transferred from the jar to a 1.5 ml micro-centrifuge tube (USA Scientific) at least once every three hours over the course of eight to 10 hours per day. This tube was then immediately placed into a −80 °C freezer (So-Low, Environmental Equipment, Cincinnati, OH) for storage until DNA extraction occurred. A new tube was used for each collection time and sample to prevent additional freeze/thaw cycles. During time points when larvae were sampled, a soil sample was also collected from the bottom of the seedling mat prior to the soil drying. For adult collection, emergence containers were checked daily and adults from each container on a given day were placed into 1.5 ml micro-centrifuge tubes. Soil was also collected from the soil surface from which adults were emerging.

### DNA extraction and quantification

Whole insects were pooled and DNA extracted using accepted methods (1–8 larvae/sample; 1–2 pupae/sample; a single adult/sample)^50^. The samples were extracted using PowerFecal® DNA Isolation Kit (MO BIO Laboratories, Inc. Catalog No. 12830-50) following the manufacturer’s protocol (https://mobio.com/media/wysiwyg/pdfs/protocols/12830.pdf) with the following modifications: one sterile 0.5 cm diameter stainless steel ball bearing was added to the Dry Bead Tube for each adult and soil sample prior to shaking; shaking time was reduced to 5 minutes for adults and 3 minutes for all other samples. DNA quality and concentration was determined for each sample by Nanodrop 2000 Spectrophotometer (Thermo Scientific, Wilmington, DE), and the DNA stored at −80 °C until further processing.

### Library construction and sequencing

All PCR and sequencing was performed at the University of Missouri DNA Core. DNA concentration was determined fluorometrically (Qubit 2.0, Life Technologies) prior to analysis. Based on results of fluorometry, all samples were normalized to a standard concentration for PCR amplification. Bacterial 16S rRNA amplicons were generated via amplification of the V4 hypervariable region of the 16S rRNA gene using single-indexed universal primers (U515F/806 R) flanked by Illumina standard adapter sequences and the following parameters: denaturation at 98 °C (3:00 mins), further denaturation at 98 °C (15 sec), annealing at 50 °C (30 sec), and extension at 72 °C (30 sec) for 25 cycles, followed by an extension at 72 °C (7 mins). Amplicons were then pooled for sequencing using the Illumina MiSeq platform and V2 chemistry with 2 × 250 bp paired-end reads, as previously described^51^.

### Informatics analysis

All informatics analyses were performed as previously described^52^ at the University of Missouri Informatics Research Core Facility. Input is typically for 2 × 350 bp reads from one of the two MiSeq machines in the DNA Core. The read pairs are joined into contigs by the program FLASH (http://bioinformatics.oxfordjournals.org/content/27/21/2957.long)^53^. The two parameters used were minimum overlap = 200 bp, and maximum overlap 225 bp, and culled if found to be short after trimming for a base quality less than 31, and those that are not joined, or are too long or short after contig formation, leaving those that are 275 to 300 nts. Cutadapt (http://journal.embnet.org/index.php/embnetjournal/article/view/200/479) was used to find and trim the primers from the 5′ and the 3′ ends, culling those contigs lacking both primers. Cutadapt was executed twice on the contig created by FLASH. The first execution was to remove the forward primer GTGCCAGCMGCCGCGGTAA, and allowed an error rate of 0.11 mismatches, a minimum length of 19 bp, and wildcard matching. Untrimmed contigs were discarded. Those not discarded were then trimmed from the 3′ end using the sequence ATTAGAWACCCBDGTAGTCC, allowing 0.1 mismatches, a minimum length of 20, and wildcard matching. Untrimmed contigs were discarded. Contigs with the expected number of errors greater than 0.5 were removed by Usearch (http://drive5.com/index.htm), and the remainder were trimmed to length 248. The contig read ids were modified so that samples could be followed throughout by using the Qiime script split_libraries_fastq.py (Python v2.7). All samples were then pooled into one FASTA file and metrics for all samples collated into one table. Contigs were clustered *de novo* into an OTU table using the uparse (http://drive5.com/uparse/) algorithm. *De novo* and reference-based chimera detection and removal was performed using Qiime v1.8^54^ software, and remaining contiguous sequences were assigned to operational taxonomic units (OTUs) via *de novo* OTU clustering and a criterion of 97% nucleotide identity. Annotation of selected OTUs was performed using BLAST^55^ against the Silva database (https://www.arb-silva.de/)^56^ of 16S rRNA sequences and taxonomy. Principal coordinate analysis and PERMANOVA testing were performed using ¼ root-transformed and non-transformed OTU relative abundance data, respectively, using Past 3.16 (https://folk.uio.no/ohammer/past/)^57^. Richness, Shannon diversity index, and Simpson diversity metrics were determined in Past 3.16 using Qiime-generated otu_biom.table files.

### Statistical analysis

Differences in raw and binned OTU richness were tested via ANOVA using SigmaPlot 12.3 (Systat Software Inc., San Jose, CA); *p* values < 0.05 were considered significant. Following the initial clustering of DNA amplicon sequences into groups sharing 97% nucleotide identity, these clusters were identified as arbitrarily numbered operational taxonomic units (OTUs), and are referred to as the raw OTUs in the current manuscript. These sequence groups were annotated against the SILVA reference database of millions of 16S rRNA sequences. Many of the aforementioned ‘raw’ OTUs annotated to the same taxonomy, and were thus binned together in the context of certain figures such as the stacked bar charts. In keeping with the conventions of the field, richness and alpha-diversity metrics were determined using the raw OTU counts. Differences in the overall composition of the different regions were tested via two- and one-way PERMANOVA of ranked Bray-Curtis or Jaccard distances using the open access Past 3.16 software package^58^, downloaded on April 2, 2016.

## Supplementary information

Supplementary Information

## Data Availability

All data are publicly available as Bioproject PRJNA422802, in the NCBI Sequence Read Archive (SRA) database.
