# Supplementary material for: Survey of bacteria associated with western corn rootworm life stages reveals no difference between insects reared in different soils
Source: Sci Rep. 2019 Oct 25;9:15332. doi: 10.1038/s41598-019-51870-x (PMC6814711; doi:10.1038/s41598-019-51870-x)
Supplement: Supplementary file 1 — Supplementary Information [file 41598_2019_51870_MOESM1_ESM.pdf]

Supplementary Information

Title:

Survey of bacteria associated with western corn rootworm life stages reveals no difference between insects reared in different soils

Authors:

Dalton C. Ludwick[1], Aaron C. Ericsson[2], Lisa N. Meihls[3], Michelle L.J. Gregory[4], Deborah L. Finke[5], Thomas A. Coudron[4,5], Bruce E. Hibbard[5,6], and Kent S. Shelby*[4,5]

Affiliations:

[1]USDA-ARS, 2217 Wiltshire Rd., Kearneysville, WV 25430, USA.
[2]Dept. of Veterinary Pathobiology, University of Missouri, Columbia, MO 65201, USA.
[3]USDA-ARS, Donald Danforth Plant Science Center, St. Louis, MO, 63132, USA.
[4]USDA-ARS, 1503 S. Providence Rd., Columbia, MO 65201, USA.
[5]Division of Plant Sciences, University of Missouri, Columbia, MO 65211, USA.
[6]USDA-ARS, 205 Curtis Hall, University of Missouri, Columbia, MO 65211, USA.

This PDF file includes:

Supplementary Table 1. Summary of richness, coverage and diversity statistics.

Supplementary Figure S1. Main effect of life stage on mean Shannon and Simpson diversity indices in western corn rootworms (A, $p < 0.001$), or the soil from which the WCR samples were collected (B, $p = 0.040$). Significant pairwise differences indicated like letters (Kruskal-Wallis one-way ANOVA on ranks with Dunn's post hoc).

Supplementary Figure S2. Principal coordinate analysis based on Jaccard similarity between bacterial communities detected in western corn rootworms (WCR) at various life stages and soil samples collected from two different sites.

Supplementary Figure S3. Number and mean relative abundance (above bars) of operational taxonomic units (OTUs) detected at increasing prevalence in adult western corn rootworm samples.

Supplementary Figure S1. Main effect of life stage on mean Shannon and Simpson diversity indices in western corn rootworms (A, $p < 0.001$), or the soil from which the WCR samples were collected (B, $p = 0.040$). Significant pairwise differences indicated like letters (Kruskal-Wallis one-way ANOVA on ranks with Dunn's post hoc).

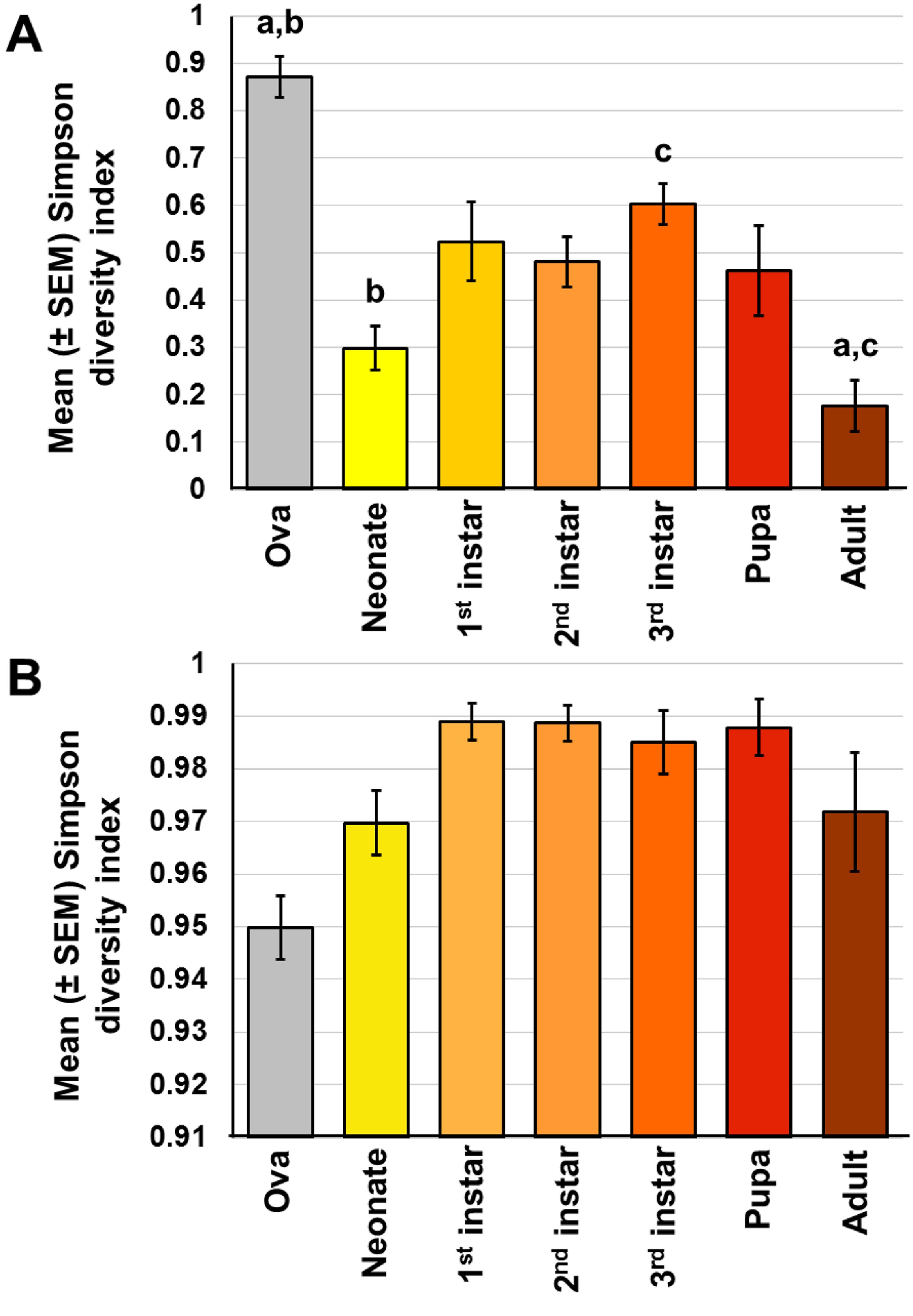

Supplementary Figure S2. Principal coordinate analysis based on Jaccard similarity between bacterial communities detected in western corn rootworms (WCR) at various life stages and soil samples collected from two different sites.

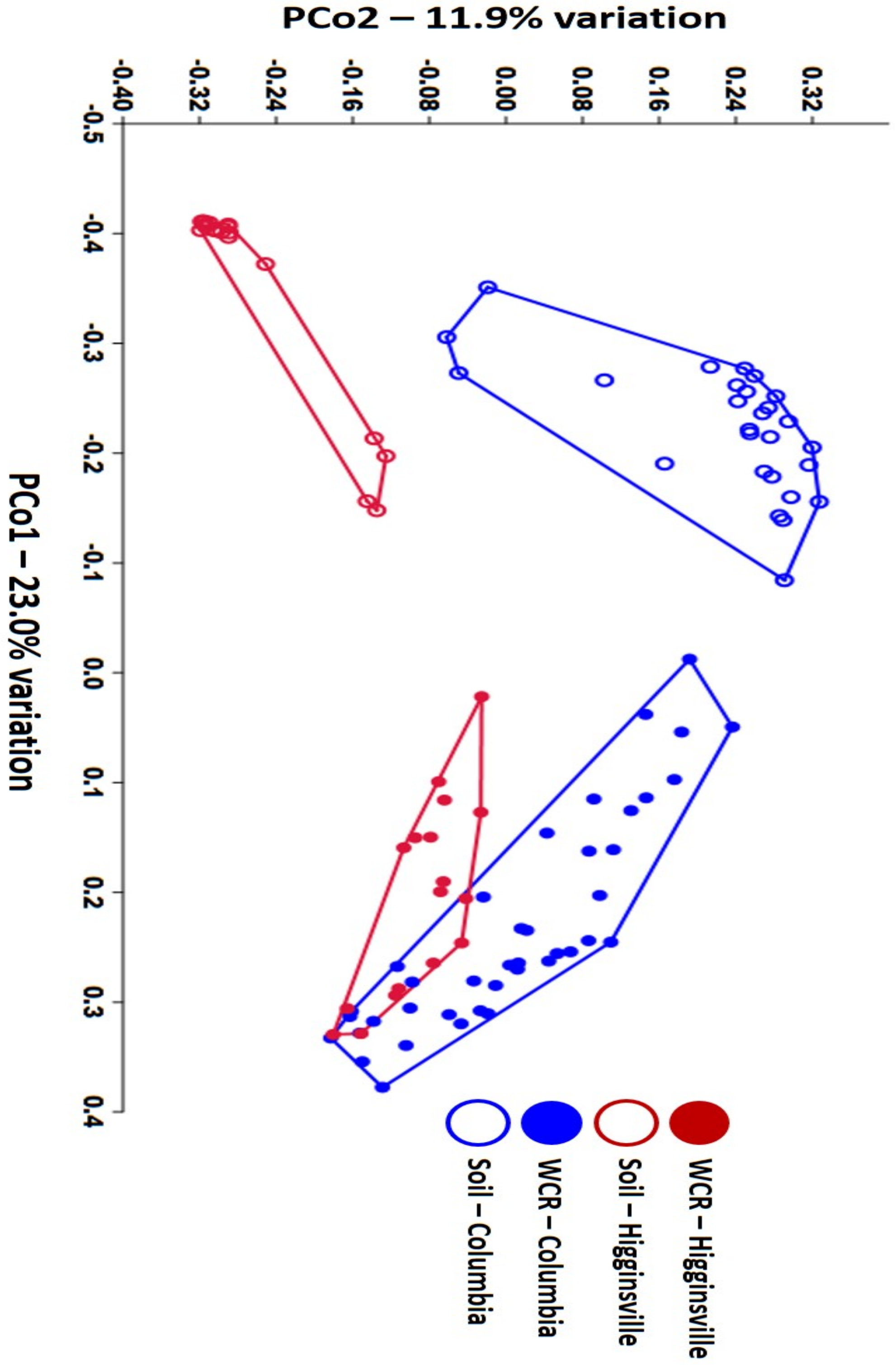

Supplementary Figure S3. Number and mean relative abundance (above bars) of operational taxonomic units (OTUs) detected at increasing prevalence in adult western corn rootworm samples.

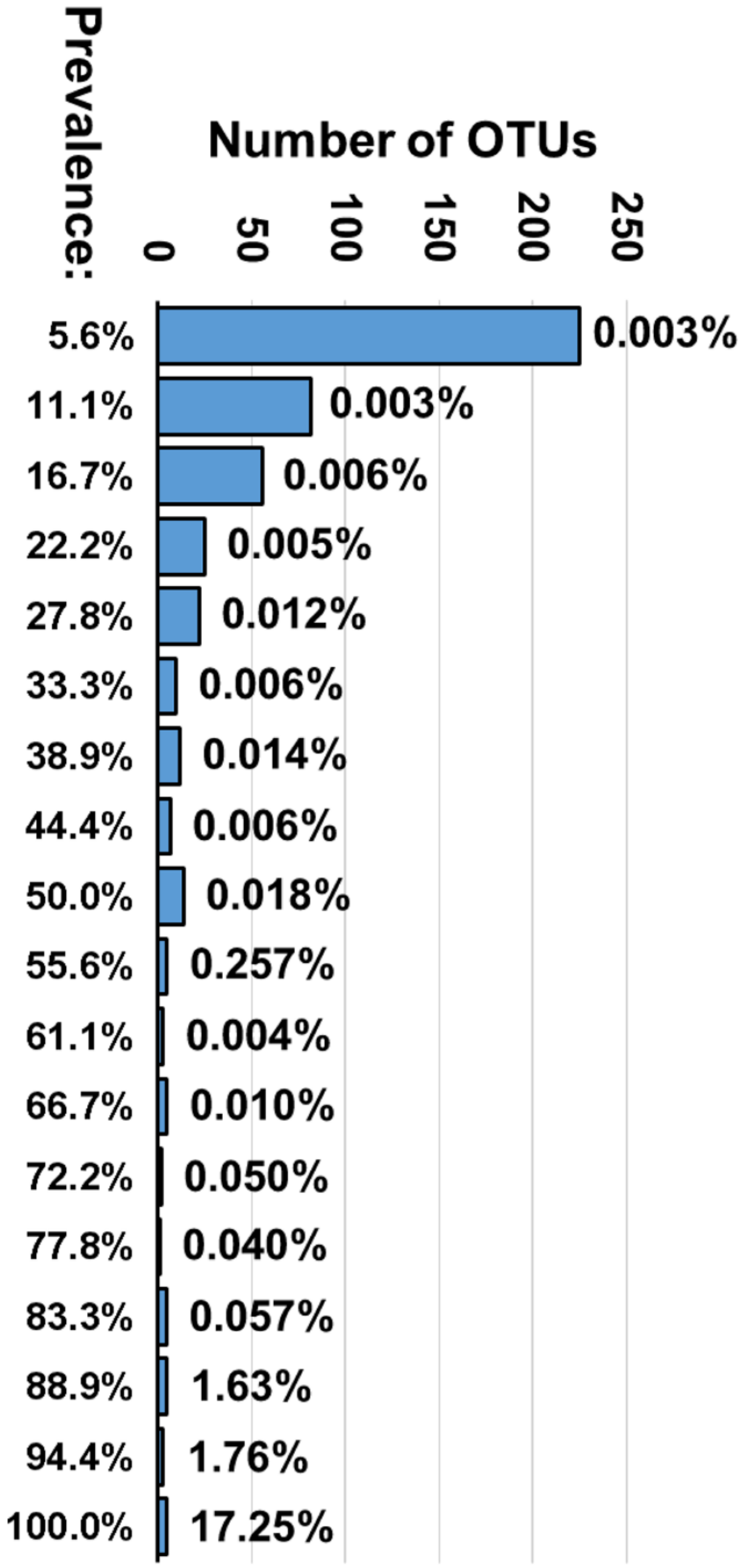

Supplementary Table 1. Summary of richness, coverage and diversity statistics.

			Read count				Good's Coverage		Richness		Shannon		Simpson	
**Sample type**	**Site**	***n***	**Mean**	**SD**	**Low**	**High**	**Mean**	**SD**	**Mean**	**SD**	**Mean**	**SD**	**Mean**	**SD**
Sterilized eggs (SE)	Columbia	6	104734	30014	44501	125466	0.997	0.002	1386	278	4.00	0.76	0.871	0.106
Neonate (N)	Columbia	6	33090	16878	15385	59963	0.997	0.001	221	74	0.88	0.22	0.299	0.115
1st instar (1st)	Columbia	4	30994	26250	9999	67530	0.997	0.002	161	60	1.44	1.02	0.550	0.255
	Higginsville	2	35501	13514	25945	45056	0.998	0.001	208	4	0.90	0.01	0.471	0.074
2nd instar (2nd)	Columbia	6	56366	24065	24504	91127	0.999	0.001	177	23	1.00	0.54	0.444	0.188
	Higginsville	3	62705	19994	42694	82682	0.999	0.000	246	61	1.09	0.01	0.557	0.019
3rd instar (3rd)	Columbia	6	76164	33551	25958	116537	0.999	0.000	274	92	1.63	0.51	0.635	0.151
	Higginsville	3	83337	23656	56292	100181	0.999	0.001	243	33	1.04	0.11	0.540	0.027
Pupa (P)	Columbia	6	59360	30073	17877	104815	0.998	0.001	305	112	1.56	0.90	0.603	0.234
	Higginsville	3	68169	16197	50327	72235	0.999	0.001	258	151	0.45	0.14	0.179	0.077
Adult F (AF)	Columbia	6	62130	36992	22709	128332	0.999	0.000	76	10	0.51	0.57	0.223	0.292
	Higginsville	3	59991	5168	54166	64025	0.999	0.000	132	15	0.49	0.62	0.192	0.277
Adult M (AM)	Columbia	6	65457	29407	33030	108808	0.999	0.000	95	20	0.24	0.29	0.113	0.201
	Higginsville	3	65154	6443	58344	71152	0.999	0.000	162	82	0.47	0.35	0.195	0.164
Soil SE	Columbia	3	92243	31011	57202	116148	0.997	0.000	997	239	4.38	0.19	0.950	0.010
Soil N	Columbia	4	81481	21164	59423	110316	0.998	0.001	1020	202	4.59	0.44	0.970	0.012
Soil 1st	Columbia	4	68838	37378	13944	97438	0.994	0.006	954	253	4.87	0.23	0.978	0.009
	Higginsville	6	80593	27574	31889	105885	0.987	0.008	4191	654	6.79	0.06	0.996	0.000
Soil 2nd	Columbia	4	109778	6790	103511	119335	0.998	0.000	1245	524	5.04	0.37	0.981	0.008
	Higginsville	4	56214	37194	16291	89597	0.976	0.018	3486	989	6.67	0.25	0.996	0.001
Soil 3rd	Columbia	2	52761	44050	54964	95669	0.990	0.008	954	269	4.85	0.69	0.977	0.017
	Higginsville	2	88387	1864	87069	89705	0.947	0.074	4443	286	6.88	0.11	0.997	0.000
Soil P	Columbia	4	71237	20904	50467	91464	0.996	0.001	1331	280	5.20	0.58	0.982	0.017
	Higginsville	2	87296	4178	84341	90250	0.941	0.087	3933	141	6.55	0.07	0.995	0.000
Soil A	Columbia	5	89537	37459	23906	112747	0.996	0.004	1099	260	4.57	0.56	0.958	0.034
	Higginsville	1	75642	n/a	n/a	n/a	0.989	n/a	3966	n/a	6.81	n/a	0.997	n/a
